# Systematic review on gene–sun exposure interactions in skin cancer

**DOI:** 10.1002/mgg3.2259

**Published:** 2023-08-03

**Authors:** Rasha Shraim, Mohamed Ziad Farran, George He, Jelena Marunica Karsaj, Lina Zgaga, Ross McManus

**Affiliations:** ^1^ Department of Public Health and Primary Care, Institute of Population Health Trinity College Dublin Dublin Ireland; ^2^ Department of Clinical Medicine, Trinity Translational Medicine Institute Trinity College Dublin Dublin Ireland; ^3^ The SFI Centre for Research Training in Genomics Data Sciences University of Galway Galway Ireland; ^4^ Department of Rheumatology, Physical Medicine and Rehabilitation Sestre milosrdnice University Hospital Center Zagreb Croatia

**Keywords:** BCC, gene–environment interaction, melanoma, SCC, skin cancer, sun exposure

## Abstract

**Background:**

The risk of skin cancer is determined by environmental factors like ultraviolet radiation (UVR), personal habits like time spent outdoors and genetic factors. This review aimed to survey existing studies in gene–environment (GxE) interaction on skin cancer risk, and report on GxE effect estimates.

**Methods:**

We searched Embase, Medline (Ovid) and Web of Science (Core Collection) and included only primary research that reported on GxE on the risk of the three most common types of skin cancer: basal cell carcinoma (BCC), squamous cell carcinoma (SCC) and melanoma. Quality assessment followed the Newcastle–Ottawa Scale. Meta‐analysis was not possible because no two studies examined the same interaction. This review was registered on PROSPERO (CRD42021238064).

**Results:**

In total 260 records were identified after exclusion of duplicates. Fifteen studies were included in the final synthesis—12 used candidate gene approach. We found some evidence of GxE interactions with sun exposure, notably, with *MC1R*, *CAT* and *NOS1* genes in melanoma, *HAL* and *IL23A* in BCC and *HAL* and *XRCC1* in SCC.

**Conclusion:**

Sun exposure seems to interact with genes involved in pigmentation, oxidative stress and immunosuppression, indicating that excessive UV exposure might exhaust oxidative defence and repair systems differentially, dependent on genetic make‐up. Further research is warranted to better understand skin cancer epidemiology and develop sun exposure recommendations. A genome‐wide approach is recommended as it might uncover unknown disease pathways dependent on UV radiation.

## Graphical abstract

The risk of skin cancer is determined by environmental factors like UV radiation, personal habits like time spent outdoors and genetic factors. This systematic review aimed to survey existing studies in gene–environment (GxE) interaction on skin cancer risk, and report on GxE effect estimates.

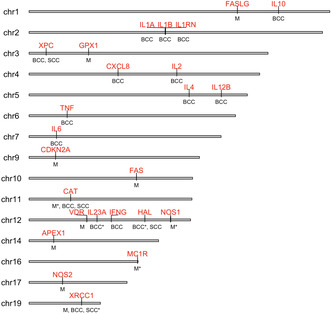

## 1 INTRODUCTION

Worldwide, skin cancer incidence is rising (Apalla et al., 2017). The three most common types of skin cancer are basal cell carcinoma (BCC), squamous cell carcinoma (SCC) and melanoma. A conservative estimate by the World Cancer Research Fund reported that approximately 1 million non‐melanoma skin cancer (NMSC) and 300,000 melanoma cases occurred globally in 2020 (World Cancer Research Fund International, 2022).

Solar ultraviolet radiation (UVR) is the most prominent environmental exposure linked with skin cancer risk (Lee et al., 2020). UVR increases skin cancer risk due to DNA damage and/or through immunosuppression (Cadet & Douki, 2018; Narayanan et al., 2010). The evidence suggests that the effect of UVR is mediated by the duration, pattern and intensity of exposure: SCC is often linked to long‐term exposure, BCC to excessive intermittent exposure and melanoma to recreational exposure and sunburn history (Apalla et al., 2017). Twin and genetic studies support the presence of a heritable, genetic component to skin cancer (Mucci et al., 2016; Robles‐Espinoza et al., 2014; Stolarova et al., 2020), estimated at 58% for melanoma and 43% for NMSC (Mucci et al., 2016). To date, over 2000 genes have been linked with skin cancer (Gene, 2004). It is reasonable to assume that genetic and environmental skin cancer risk factors are interdependent (Simonds et al., 2016).

We hypothesize that gene–environment (GxE) interactions may offer further insight in understanding the aetiology of skin cancer and point to primary and secondary prevention solutions. By definition, GxE indicates a significant deviation from the expected combined effect of the genetic and the environmental factors (see Thomas, 2010). In this systematic review, we review and summarize the research on GxE interactions in three types of skin cancer, melanoma, BCC and SCC, with a focus on UVR.

## 2 METHODS

### 2.1 Search strategy

We searched the Embase, Medline (Ovid) and Web of Science (Core Collection) databases without any language restrictions from inception until 22 June 2022. The key terms in the search included skin cancer, melanoma, carcinoma and GxE interactions. The full search strategy for each database is available in Material S1. This systematic review was conducted according to the Preferred Reporting Items for Systematic reviews and Meta‐Analyses (PRISMA) statement (Page et al., 2021). The protocol was registered with PROSPERO (CRD42021238064).

### 2.2 Inclusion and exclusion criteria

Studies were included if they investigated the risk of skin cancer (melanoma, SCC or BCC) measured either by odds ratio, risk ratio or hazard ratio. Studies that reported on pre‐cancer conditions such as pre‐cancerous skin lesions were not included. The exposure of interest was GxE interaction, restricted to natural environmental factors, predominantly sun exposure. Observational studies (cohort, case–control, cross‐sectional) were eligible, as long as they reported an analysis of GxE interactions. Studies that only reported on main effects, that is, genetic factors or environmental factors alone, were excluded. We also excluded studies that reported on prognosis, survival rate or other outcomes.

### 2.3 Study selection

Covidence was used to facilitate the study selection and extraction of data (VH Innovation, n.d.). After the removal of duplicates, titles and abstracts were screened by two independent researchers (S.H. and M.Z.F.) and disagreements were resolved in discussion with a third researcher (R.S.). Full texts were obtained for eligible studies and assessed. Relevant review articles were excluded but screened for references; the reference lists of eligible studies were similarly screened.

### 2.4 Data extraction and quality assessment

From each eligible study, two researchers (R.S. and R.M. or J.M.K.) extracted and recorded the following data: publication details (including corresponding author, journal, publication date), study details (including design, aim, sample size, population characteristics, environmental exposure measures) and outcome measures (including skin cancer type(s), effect size estimates). Quality assessment was performed using the Newcastle–Ottawa Scale for cohort or case–control studies as appropriate (Wells et al., 2013).

We summarize and present the effects of GxE on skin cancer based on the current literature and describe the different study setups, including the various cohorts, genetic variants, environmental exposure proxies and effect measures used to date. After reviewing the available GxE studies in skin cancer, we found no two studies that investigated the same interaction (i.e. the same genetic factor with a particular environmental exposure) and were thus unable to conduct a meta‐analysis of GxE effects at this time.

## 3 RESULTS

### 3.1 Study selection

**FIGURE 1:**
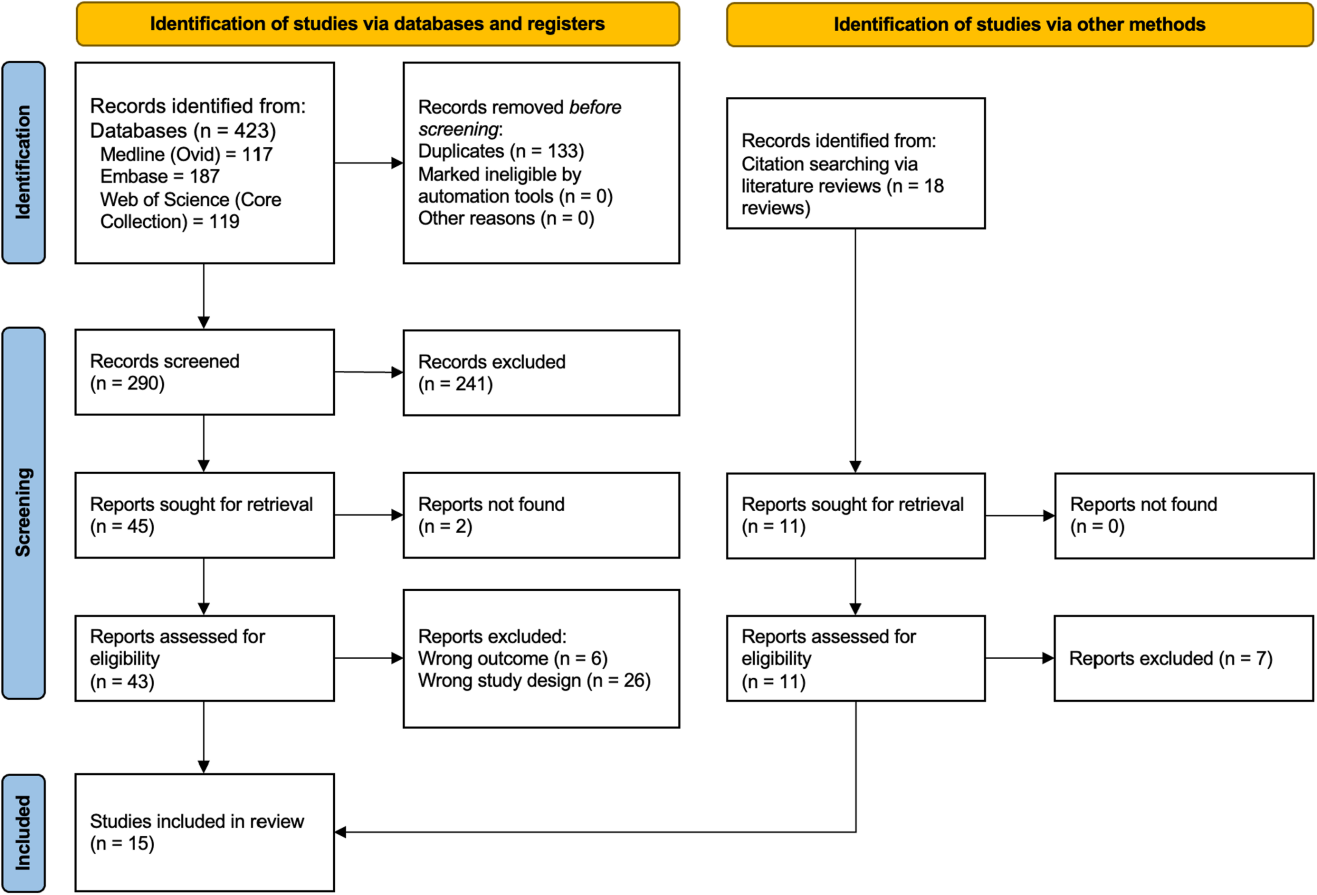
PRISMA flow diagram of study selection.

The search process is outlined in Figure 1. We identified 290 records after exclusion of duplicates. Based on relevance and eligibility criteria, 15 studies were included in the final synthesis.

### 3.2 Study characteristics

Nine studies reported on melanoma, seven on BCC and four on SCC (one study reported on all three cancers, and three studies reported on BCC and SCC). Case sample size varied widely from 420 melanoma cases (Olsen et al., 2020) to 17,187 BCC cases (Chahal, Lin, et al., 2016; He et al., 2010; Lin et al., 2017). Many of the included studies used the same sample cohorts: cancer patients at the University of Texas MD Anderson Cancer Center (Li et al., 2007; Li, Larson, et al., 2006; Li, Liu, et al., 2006), the Nurses Health Study (Chahal, Wu, et al., 2016; He et al., 2010; Lin et al., 2017), the Health Professionals Study (Chahal, Wu, et al., 2016; Lin et al., 2017), the New Hampshire Health Study (Nelson et al., 2002, 2005; Welsh et al., 2008), and GEM (Berwick et al., 2011; Kricker et al., 2010; Mandelcorn‐Monson et al., 2011). All of the participants were of white European ancestry. Details of the age and sex distribution of the participants were limited or unavailable in some studies. Generally, participants tended to be middle aged and male.

### 3.3 Risk of bias in studies

The majority of the included papers had a low risk of bias (Table S1). However, two of the included studies were only published as a conference abstract (Ng et al., 2011) or letter to the editor (Berwick et al., 2011); hence some of the information about the study design was not given, leading to a high risk of bias score.

### 3.4 Study design

Table 1 includes a summary of the key characteristics of included studies. Only Olsen et al. (2020) used a cohort study design whereas all others were case–control studies. Only four studies used direct estimates of sun exposure based on measured UV (Chahal, Wu, et al., 2016; He et al., 2010; Kricker et al., 2010; Lin et al., 2017). Others used proxies of sun exposure such as lifetime sunburns or sunny holidays. All studies adjusted for age and sex with some additionally adjusting for recruitment centre, sun exposure history, cancer history, population stratification, pigment scores and others. For the genetic factor, while Olsen et al. (2020) reported on PRS and Lin et al. (2017) on over 2500 SNPs across VDR sites, the remaining reported on one or a few candidate genes. While a number of studies used the same study sample, the approach to GxE was varied.

**TABLE 1:** Summary of the included studies. Sample characteristics are provided for the GxE analysis only, which may be different from the main analysis.

Cohort	Citation	*N* cases	*N* controls	Gene	Environment	GxE findings	Direction of the interaction effect [95% CI]
Hungary, Romania and Slovakia (2002–2004): European individuals, age ~60 (25–75), ~52%F	Rizzato et al. (2011)	529 BCC	532	14 SNPs in 10 cytokine genes, 3 previously reported SNPs	Sun exposure	SNP × sun exposure: no significant evidence of interaction; evidence of association with tanning and *IL1B* rs1143627 (*p*‐trend = 0.0019), with blistered skin and *TNF* rs1800629 (*p*‐trend = 0.0005)	No statistically significant evidence of interaction
Minnesota Skin Health Study participants	Ng et al. (2011)^a^	924 M	813	*MC1R* variants	Sun exposure: total hours, hours of outdoor activity, lifetime sunburns	*MC1R* × sun exposure: no significant evidence of interaction	No statistically significant evidence of interaction
Qskin Sun and Health Study (2011): European‐ancestry Australians, age 57 (SD 7.9, 40–69), 55%F	Olsen et al. (2020)	420 M	15,200	PRS (participants grouped into risk tertiles based on the distribution of the entire sample)	UV radiation exposure: residence, birth country; personal exposure; proxies of high exposure, for example, actinic lesions, NMSC	PGS × UV exposure: evidence of interaction with country of birth (*p* = 0.03) and number of actinic lesions (*p* = 0.03) but not youth sunburns, sunbed use or skin cancers excised (*p* > 0.2)	Risk of melanoma *exacerbated* for participants born in Australia who have high genetic risk (PRS tertile 3): HR = 3.16 [1.39–7.22] compared to those with low risk (PRS tertile 1): HR = 0.88 [0.38–2.04]. Risk of melanoma *ameliorated* for participants with ≥6 actinic lesions with high genetic risk: HR = 1.97 [1.14–3.40] compared to those with low genetic risk (HR = 6.74 [2.41–18.8])
M. D. Anderson Cancer Center (1994–2004): non‐Hispanic white hospital patients from the USA	Li, Larson, et al. (2006), Li, Liu, et al. (2006)^b^	602 M	603	*FAS* and *FASLG* combined genotypes (FAS‐1377G>A, FAS‐670A>G, FASLG‐844T>C, FASLG‐IVS2nt‐124G>A)	Sun exposure history: tanning ability, lifetime sunburns with blistering, freckling as a child	*FAS* and *FASLG* genotype × sun exposure history: no evidence of interaction (evidence of interaction with eye colour, *p* = 0.038)	Risk of melanoma is *ameliorated* in participants with high genetic risk (four to eight risk alleles) and blue eye colour: OR = 3.98 [2.81–6.65], compared to those with low genetic risk (0–3 risk alleles) and blue eye colour: OR = 4.63 [3.10–6.92]
	Li, Larson, et al. (2006), Li, Liu, et al. (2006)^c^	—	—	*APE1* and *XRCC1* combined genotype	—	*APEX1* & *XRCC1* genotype × sun exposure history: no evidence of interaction	No statistically significant evidence of interaction
	Li et al. (2007)	—	—	*nNOS* and *iNOS* SNPs (nNOS‐84, nNOS 276, iNOS Ex16, iNOS 974)	—	SNP × sun exposure: evidence of interaction of *nNOS* combined genotype (−84 GG/276 [CT + TT]) with having moles (*p* = 0.002) and sunburns (*p* = 0.017)	Risk of melanoma *ameliorated* in participants with moles and ‐84 GG/276 (CT + TT)genotype: OR = 2.33 (1.51–3.60), compared those with moles and other genotypes: OR = 4.92 (3.32–7.30). Risk of melanoma *exacerbated* in participants with ≥1 lifetime sunburns and ‐84 GG/276 (CT + TT) genotype: OR = 1.14 [0.72–1.79], compared ≥1 sunburns and other genotypes: OR = 1.17 (0.78–1.74) but the added risk of sunburns is greater in the −84 GG/276 (CT + TT) genotype group (OR with no sunburns = 0.36)
GEM (2000–2003): European ancestry participants from Australia, Canada, Italy, USA, age ~50s–60s, 43%F	Kricker et al. (2010)	1018 MPM	1875 SPM	MC1R gene (alternative = Any R includes R151C, R160W, D294H and D84E variants; reference = no R includes r in the absence of R and con/con)	UV: estimated as the annual average erythemally weighted UV at place of residence at birth and in each decade	*MC1R* × sun exposure: some interaction with beach and water activities (*p* = 0.08) but not lifetime UV or early‐life UV (*p* = 0.93 and 0.81, respectively). Stratified by body site, early‐life UV was significant on head and neck (*p* = 0.01) while water activities only on other body sites (*p* = 0.08)	Risk of MPM *ameliorated* on head and neck in participants with high early‐life ambient UV and any R allele: OR = 3.03 [1.46–6.30], compared to high UV and no R allele: OR = 4.23 [1.76–10.20] Risk of MPM *ameliorated* on other body sites in participants with any water activities and any R allele: OR = 1.39 [1.02–1.91], compared to no water activities and no R allele: OR = 2.02 [1.37–2.98]
	Berwick et al. (2011)^d^	2469 SPM, 1207 MPM	2373	*CDKN2A* (exons 1a, 2 and 3)	Sun exposure: early‐life exposure, sunny holidays, beach and waterside exposure, lifetime burns	*CDKN2A* x sun exposure: no significant evidence of interaction	No statistically significant evidence of interaction
	Mandelcorn‐Monson (2011)	1138 MPM	2151 SPM	*VDR FokI* and *Bsml* polymorphisms	Sun exposure: early lifetime UV exposure, sunny vacations, beach and waterside exposure	*VDR* x sun exposure: no significant evidence of interaction	No statistically significant evidence of interaction
Nurses Health Study (1976): Individuals from the USA, age 30–55, 100%F; Health Professionals Study (1986): European‐ancestry participants from the USA, 100% male, age 40–75; 23&Me	He et al. (2010) (Nurses Health Study only)	218 M, 285 SCC, 300 BCC	870	*GPX1* and *CAT* genes (CAT C‐262 T polymorphism, reference = CC, alternative = TC + TT)	Sun exposure: personal exposure and solar radiation measures from weather station nearest to residence	SNP × sun exposure: evidence of interaction between CAT C‐262 T polymorphism and history of severe sunburns on melanoma (*p* = 0.008)	Risk of melanoma *exacerbated* in participants with high history of severe sunburns and TC + TT genotype: OR = 1.73 [1.02–2.92], compared to those with high sunburns and CC genotype: OR = 1.03 [0.63–1.69]
	Chahal, Lin, et al. (2016); Chahal, Wu, et al. (2016)	17,187 BCC	287,054	31 GWAS loci	UV exposure based on residence location in 1986	SNP × UVB: no significant evidence of interaction with sunburn	No statistically significant evidence of interaction
	Lin et al. (2017)	—	—	2540 independent SNPs across *VDR* binding sites	Cumulative lifetime sun exposure: mean solar radiation at past and present residence from a UV database and questionnaire	SNP × sun exposure: evidence of interaction between sun exposure level and rs79824801 on BCC risk (*p* = 0.02)	No statistically significant evidence of interaction
New Hampshire Health Study (1993–2000): Newly diagnosed European‐ancestry patients in the USA, average age ~60, ~40%F	Nelson et al. (2002)	499 BCC, 246 SCC	431	*XRCC1* arg399gln polymorphism	Ionizing radiation, sunburns	*XRCC1* × sun exposure: no direct evidence of interaction but the difference in models with and without the sunburn interaction terms was statistically significant for SCC (*p* < 0.02)	Limited evidence of statistically significant interaction but reported risk of SCC is *exacerbated* in participants with any gln variant and ≥3 lifetime sunburns, for gln/gln: OR = 6.8 [2.4–19.2], compared to arg/arg wild type and ≥3 sunburns: OR = 1.5 [0.9–2.5]
	Nelson et al. (2005)	732 BCC, 572 SCC	613	*XPC PAT* allele	Average hours sun exposure per week, sunburn history or history of ionizing radiation therapy	*XPC* PAT polymorphism × sun exposure: no significant evidence of interaction in BCC or SCC	No statistically significant evidence of interaction
	Welsh et al. (2008)	914 BCC, 702 SCC	848	*HAL* locus as a binary variable with dominant inheritance	Sun exposure: sunburn history	*HAL* genotype × sun exposure: evidence of interaction with severe sunburns (BCC *p* = 0.040, SCC *p* = 0.018)	Risk of BCC is *exacerbated* with any G variant and ≥4 sunburns, in GG genotype: OR = 2.7 (1.3–5.8), compared to AA genotype: OR = 1.3 (1.0–1.7). Risk of SCC is similarly *exacerbated* with any G variant, in GG genotype: OR = 3.4 (1.6–7.5), compared to AA genotype: OR = 1.5 [1.1–1.9]

*Note*: Some information has been omitted for readability: see File S2 for all effect measures and Table S2 for genetic quality control and covariates.

Abbreviations: BCC, basal cell carcinoma; HR, hazard ratio [95% confidence interval]; HWE, Hardy–Weinberg equilibrium; LD, linkage disequilibrium; M, Melanoma; MAF, minor allele frequency; MPM, multiple primary melanoma; OR, odds ratio; PRS, polygenic risk score; SCC, squamous cell carcinoma; SPM, single primary melanoma.

^a^ Conference abstract (#8504 in ASCO Annual Meeting 2011).

^b^ Pharmacogenetics & Genomics.

^c^ Carcinogenesis.

^d^ Letter to the Editor. doi: 10.1038/jid.2011.235 and #126 in Melanoma 2010 Congress.

### 3.5 Cohorts

Six cohorts or sample populations were analysed by two or more of the included studies: The Nurses Health Study recruited middle‐aged female nurses in 1976 in the United States; the Health Professionals Follow‐Up Study recruited middle‐aged male health professionals in 1986 and was designed to complement the Nurses' Health Study; 23andMe is a biotechnology company that offers DNA testing and makes genetic data available for research (participants can opt in/out); Genes, Environment, & Melanoma (GEM) is a population‐based case–control study with participants from North America, Europe and Australia recruited from 1998 to 2003; the New Hampshire Health Study recruited participants through dermatologists and pathology laboratories with controls from the State Department of Transportation and Center for Medicaid and Medicare Services between 1993 and 2000; and finally, the University of Texas M.D. Anderson Cancer Center patients were recruited at the hospital between 1994 and 2004. Additionally, data were used from hospital patients in Hungary, Romania and Slovakia recruited between 2002 and 2004, the QSkin Sun and Healthy Study cohort randomly sampled in Queensland, Australia in 2011 and the Minnesota Skin Health Study in one study each. All studies used healthy controls except the two studies that used the GEM population—their case–control comparison used multiple primary melanoma (MPM) versus single primary melanoma (Kricker et al., 2010; Mandelcorn‐Monson et al., 2011).

### 3.6 Interaction findings

**FIGURE 2:**
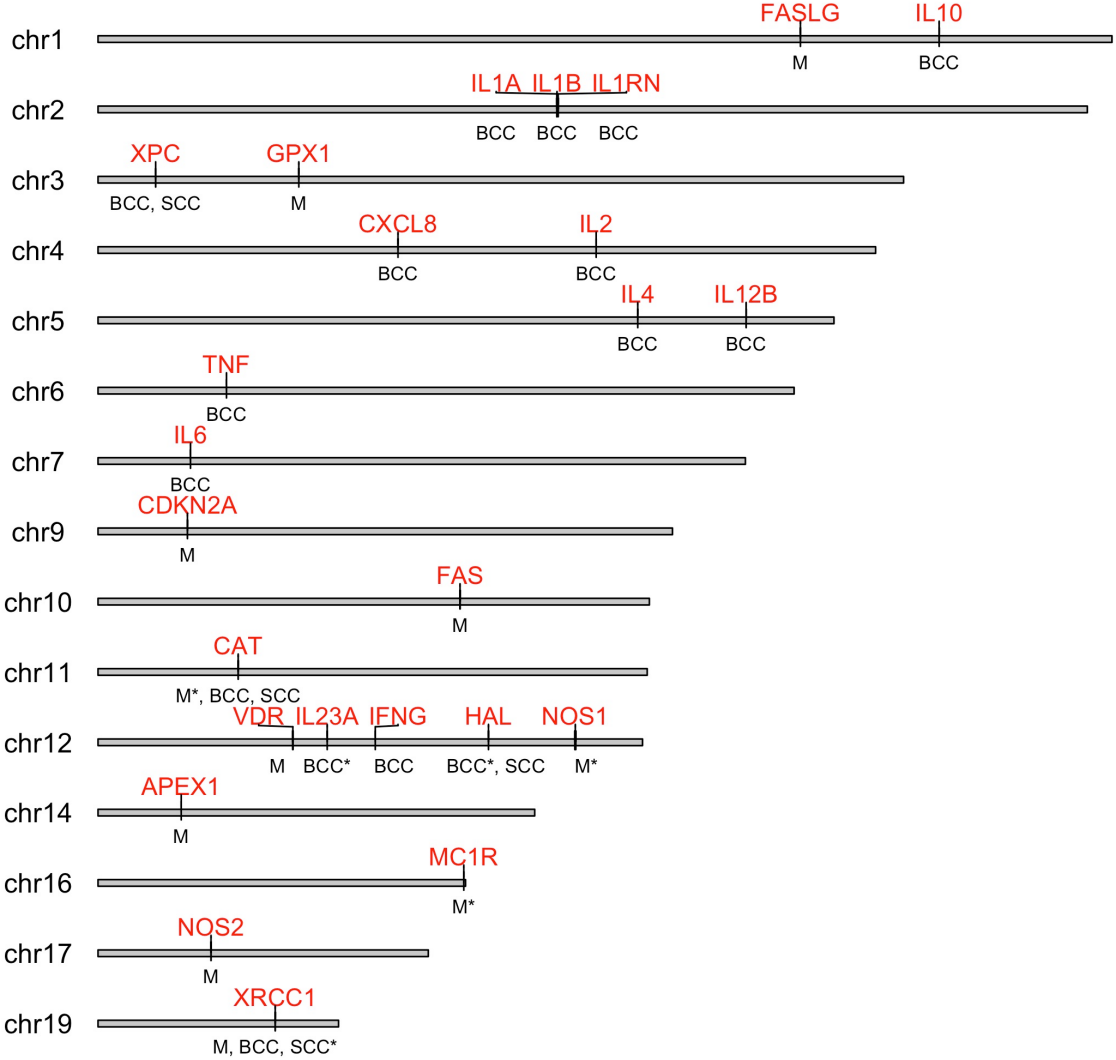
Chromosomal map showing the genes assessed for gene–environment interactions from the studies included in this review. Chromosomes with no variants tested are not shown. Genes are shown in red with the corresponding cancer types shown below, an asterisk indicates significant interaction. Due to differences in names used across the included studies, we updated gene names according to HGNC nomenclature: *APEX1* (*APE1*), *NOS1* (*NOS*, isoforms *nNOS*, *iNOS*), *CXCL8* (*IL8*), *IL12B* (*IL12*). Twenty‐eight SNPs used for polygenic risk score, PRS, are not shown (see table S1 in Olsen et al. 2020 for the full list).

The evidence for GxE interaction in skin cancer is limited. Below is a brief summary of the results by skin cancer type. Figure 2 summarizes the genes that were tested across the three skin cancer types.

#### 3.6.1 Melanoma

Melanoma was the most studied skin cancer type in this context (Berwick et al., 2011; He et al., 2010; Kricker et al., 2010; Li et al., 2007; Li, Larson, et al., 2006; Li, Liu, et al., 2006; Mandelcorn‐Monson et al., 2011; Ng et al., 2011; Olsen et al., 2020). Of nine studies, we included, only four found significant evidence of interaction (He et al., 2010; Kricker et al., 2010; Li et al., 2007; Olsen et al., 2020). Genes tested included *FAS* (OMIM: 134637), *FASLG* (OMIM: 134638), *APEX1* (OMIM: 107748), *XRCC1* (OMIM: 194360), *NOS1* (OMIM: 163731), *MC1R* (OMIM: 155555), *GPX1* (OMIM: 138320), *CAT* (OMIM: 115500), *CDKN2A* (OMIM: 600160) and *VDR* (OMIM: 601769). Olsen et al. (2020) used PRS calculated from summary statistics from a melanoma GWAS meta‐analysis (Law et al., 2015; see table S1 in Olsen et al., 2020) for full list of 28 SNPs used for the PRS). There was significant evidence of interaction of PRS with country of birth (Australia, *p* = 0.03) and with history of actinic lesions (*p* = 0.03), as proxies of high sun exposure. He et al. (2010) found evidence of interaction between the *CAT* C‐262T polymorphism and history of severe sunburns (*p* = 0.008). Of the two NOS isoforms examined (*neuronal* NOS and *inducible* NOS), Li et al. (2007) found evidence of interaction between *NOS1* (*nNOS*) and the lifetime number of sunburns with blistering (*p* = 0.017).

Kricker et al. (2010) and Ng et al. (2011) both reported on the *MC1R* gene but it was not possible to perform a meta‐analysis on their results due to differences in design and data not being reported: Kricker et al. (2010) uses independent UV data at place of residence while in their conference abstract Ng et al. (2011) use outdoor activity and sunburns. Ng et al. (2011) did not find significant evidence of interaction in *MC1R*. Comparing single‐site versus MPM, Kricker et al. (2010) only found significant evidence of interaction when stratified by body site (head and neck *p* = 0.01).

#### 3.6.2 Basal cell carcinoma

Seven studies reported on BCC (Chahal, Wu, et al., 2016; He et al., 2010; Lin et al., 2017; Nelson et al., 2002, 2005; Rizzato et al., 2011; Welsh et al., 2008). Analysed genes included *XRCC1*, *XPC* (OMIM: 613208), *HAL* (OMIM: 609457), *GPX1* (OMIM: 138320), *CAT*, *VDR*, 10 cytokine genes and 31 GWAS loci. Only two studies found direct evidence of interaction: rs7297245 with sunburn history (*p* = 0.04, *HAL*) and rs79824801 with cumulative lifetime sun exposure (*p* < 0.02, downstream of *IL23A*) (Lin et al., 2017; Welsh et al., 2008). Cumulative sun exposure in Lin et al. (2017) combined mean solar radiation values from an independent UV database at past and present residences with questionnaire information.

#### 3.6.3 Squamous cell carcinoma

Only four studies investigated SCC (He et al., 2010; Nelson et al., 2002, 2005; Welsh et al., 2008), three of which were based on the New Hampshire cohort (Nelson et al., 2002, 2005; Welsh et al., 2008). The reported genes included *XRCC1*, *XPC*, *HAL*, *GPX1* and *CAT*. Nelson et al. (2002) reported significant interaction with *XRCC1* (*p* < 0.02) and Welsh et al. (2008) reported significant interaction with *HAL* (*p* = 0.018, also significant in BCC), both using sunburn as the exposure.

## 4 DISCUSSION

Having systematically reviewed the literature to date, we found some evidence of gene–environment interactions with sun exposure in skin cancer. Most notably, with *MC1R*, *CAT* and *NOS1* genes in melanoma, *HAL* and *IL23A* genes in BCC and *HAL* and *XRCC1* genes in SCC.

UVR exposure might induce skin cancer through two possible mechanisms (Godic et al., 2014). The first implicates DNA damage and oxidative stress. Interestingly, all of the interacting genes in melanoma appear linked to this pathway. Melanocortin 1 receptor (*MC1R*) encodes the receptor protein for melanocyte‐stimulating hormone (MSH) and is involved in determining skin and hair pigmentation. It affects endogenous protection and sun sensitivity by reducing the UVR that penetrates the skin. The unabsorbed UVR contributes to oxidative stress, but harmful effects are managed through a complex antioxidant defence system (Godic et al., 2014). *CAT* encodes catalase, a key antioxidant enzyme in the body's defence against oxidative stress. Sun exposure has been shown to suppress catalase activity in a dose‐dependent manner and may increase the risk of oxidative damage (Hellemans et al., 2003). Nitric oxide synthase (*NOS1*) encodes the protein responsible for synthesizing nitric oxide (NO), which acts as a mediator in processes like vasodilatation, neurotransmission and immune response. Interestingly, NO can be liberated from nitrates and nitrites in the skin following UVA exposure, independently of NOS enzyme activity (Liu et al., 2014; Suschek et al., 2010).

The second mechanism suggests that UV exposure may induce skin cancer via immunosuppression, and failure to detect and remove cancerous cells (Godic et al., 2014). Most NMSC GxE genes reported to date are associated with this pathway. *HAL* encodes histidase, an enzyme involved in histidine catabolism. Histidase converts excess histidine in the skin to *trans*‐urocanate (UCA), (GeneCards, 2022) which is then converted to *cis*‐UCA under the influence of UV light (Brosnan & Brosnan, 2020). Experimental evidence suggests that cis‐UCA plays a key role in systemic UV‐induced immunosuppression (Welsh et al., 2008). *IL23A* encodes interleukin‐23 subunit alpha that associates with IL12B to form the pro‐inflammatory cytokine IL‐23 (Oppmann et al., 2000). Majewski et al. (2010) have shown that IL‐23 is important in reducing UV‐induced DNA damage and inhibiting UV‐induced regulatory T cells in an acute UV‐induced immunosuppression model (Yan et al., 2018). Finally, X‐ray repair cross complementing 1 (*XRCC1*) encodes a scaffolding protein involved in various DNA damage repair processes, leaving permanent mutations when the damage is too extensive and the capacity of the repair system exhausted.

Our findings suggest that the differential genetic predisposition is put to test under the excessive UVR exposure. Overall, higher sun exposure, measured directly or through proxies like history of sunburns, increases the risk of skin cancers. However, the available evidence for GxE indicates that this effect may be ameliorated or exacerbated depending on the background genetic risk. Generally speaking, GxE effects may provide an opportunity for an intervention when the environmental exposure is modifiable. In this context, this might involve a focus on primary prevention in individuals with greater genetic predisposition to sun exposure‐induced damage, such as the avoidance of excessive sun exposure or exogenous supplementation of antioxidants before UV exposure to boost antioxidant defence (Godic et al., 2014). On the other hand, GxE may provide an opportunity to carefully consider a more balanced approach to sun exposure that also considers benefits of carefully tailored exposures in individuals at decreased risk. Additionally, our findings are in line with a 2016 systematic review of GxE in all cancer, which identified *XRCC1* and *VDR* as two of the most frequently reported genes with interactions and sun exposure as one of the most commonly reported environmental exposures (Simonds et al., 2016).

As the public health burden of skin cancer continues to rise (Gordon & Rowell, 2015), studying these interactions can elucidate the ‘population‐attributable’ effects of environmental exposures, such as UVR and allow us to tailor public health advice to the population or individual's genetics (Hunter, 2005). Complete avoidance of sun exposure might negatively impact health, for example by promoting vitamin D deficiency (O'Sullivan et al., 2019) or increased blood pressure (Weller et al., 2020). For this reason, we advocate that personalized healthy sun exposure should be sought, and evidence to inform this pursued.

Many of the genes identified here have a known relevance to skin conditions. This is unsurprising given that 12 of the 15 included studies used the candidate gene approach. We note the absence of any genome‐wide GxE interaction study on skin cancer. Consequently, the scope of investigation is limited to genes *already associated with skin cancer*. Thus, we are losing out on one of the advantages of the GxE approach: the potential to uncover cancer‐causing, environmentally dependent genetic factors that may have been missed in genetic‐only association studies. This is also in line with Simonds et al. (2016) systematic review of GxE in all cancers, in which none of the 272 included studies used a genome‐wide interaction study approach and the majority relied on the candidate gene approach. This review also identified similar limitations related to sample size and the reporting of results as discussed below; though a notable improvement is that most of the included studies reported effect estimates as well as *p*‐values (File S2; Simonds et al., 2016).

The available evidence supports GxE in skin cancer but further research is warranted. Future research on this topic would benefit from following the recommendations laid out in Dunn et al. (2011) and Shraim et al. (2022). In line with those recommendations, this review highlights the need for developing guidelines for GxE research and reporting. The limitation of small sample sizes can be partly overcome by meta‐analyses, but this requires detailed and accurate reporting of analyses performed in independent cohorts, including: the chosen model and its parameters, description of environmental exposure, the interaction term tested, effect size measurements and significance thresholds. Published guidelines such as STROBE (STrengthening the REporting of OBservational studies in Epidemiology) and STREGA (Strengthening The Reporting of Genetic Association studies) can further inform reporting on methods and results (Little et al., 2009; von Elm et al., 2008). For reporting an effect measure such as OR or HR, we recommend reporting both stratified and joint results, where low sun exposure is first taken as the referent for each genotype group separately, followed up by analysis that takes low sun exposure/low risk genotype as the referent and reports results for all other groups (e.g. as in Kricker et al., 2010). By definition, a statistically significant interaction means that the impact of sun exposure on skin cancer risk will be modified by the genetic profile so this simplifies the interpretation of the results for the reader and portrays the direction of the effect clearly.

The collection of accurate information on sun exposure and sunburn is problematic. Issues include the limitations associated with self‐reporting, crude approximations (e.g. using country of birth to capture sun exposure‐related events) and vague definitions (e.g. what constitutes a severe sunburn). It seems probable that exposures over very long periods of time—if not the entire lifetime—contribute to skin cancer risk, and their changing nature and importance over the life course is difficult to capture. The choice of exposure varied widely among the included studies: only four studies used independent UV measures based on place of residence (Chahal, Wu, et al., 2016; He et al., 2010; Kricker et al., 2010; Lin et al., 2017). The remaining studies relied on questionnaires about personal sun exposure, including hours of outdoor activity, lifetime sunburns and beach vacations. Sun exposure can vary dramatically by time of the year and geographical location. For example, erythema values are higher by an order of magnitude in January in the south of the United Kingdom compared to the north (Kazantzidis et al., 2015; Kelly et al., 2016; O'Sullivan et al., 2018). Thus, participants who respond similarly to a question on time spent outdoors may be receiving notably different UV doses depending on their geographical location. The inverse is also true: two individuals may reside at the same place, but different habits, clothing preferences or time spent outdoors can lead to divergent exposures. The advantage of using independent UV data is in that it ensures that ambient exposure is comparable across studies. However, individual's exposure will be determined by both, environmental factors and personal characteristics and choices, and we should seek to capture both. For instance, where direct UV measures reflect participants' ambient UV dose, other measures such as history of sunburns, sunny holidays or outdoor time could also be taken into account. These can be discretely categorized, as in many of the included studies, into high/low groups or discrete bins to investigate dose–response relationships and mitigate the impact of the measurement error.

The existing heterogeneity is further inflated when different studies are being compared, due to the inherent between‐population differences, including the level of skin pigmentation, sunburn prevalence, solar UVR and the popularity of artificial tanning. Given that skin cancer types have been associated with specific patterns of sun exposures (Apalla et al., 2017), it is important to include these variables in the analysis.

Finally, UV exposure is not entirely independent of genetic factors: for example, lighter skinned individuals tend to take measures to reduce sun exposure (Apalla et al., 2017; Gallagher et al., 1995). Where possible, Fitzpatrick's sun reactive skin type, pigmentation and the presence of nevi should also be evaluated and included. In addition to the individual's history of sun exposure and sunburns (Apalla et al., 2017), risk is also affected by family history: (Asgari et al., 2015; Berlin et al., 2015; Robles‐Espinoza et al., 2014) relatives of cancer patients on the one hand experience a protective effect due to changed health‐oriented attitudes (Small et al., 2019), but on the other they might have increased risk due to shared sun exposure patterns (Soura et al., 2016). Therefore, future studies should take ambient environmental and personal exposure into account, where the former refers to independent measures of UV and the latter to individual factors like history of sunburns and skin characteristics.

### 4.1 Other environmental exposures

It is worth noting two other environmental exposures that appeared in several studies but did not meet our inclusion criteria. First, several studies from Bangladesh where poor quality drinking water leads to high arsenic exposure found evidence of gene–arsenic interactions, but only pre‐cancerous skin lesions were examined (Breton et al., 2007; Kibriya et al., 2017; McCarty et al., 2007; Pierce et al., 2013; Seow et al., 2015). Arsenic exposure is a global public health concern, which warrants further research, including into skin cancer outcomes. Second, we excluded studies on diet because they did not fit our criterion of “natural environmental exposure”, but we note that some evidence of interaction was found. For example, He et al. (2010) found evidence of gene–carotenoid intake interaction and Marley et al. (2022) of gene–citrus consumption interaction on the risk of melanoma.

### 4.2 Strengths and limitations

We systematically reviewed the available literature across three databases and evaluated the available evidence of GxE in skin cancer. Record assessment and data extraction were performed by two independent reviewers, which minimized the risk of excluding relevant information. One limitation was that we could not carry out a meta‐analysis. Like any review, the findings are dependent on the available studies and their quality. Several patient cohorts were reused across multiple studies, and thus their findings cannot be treated as independent. The sample size of eligible studies was small in the context of genetic research and therefore likely underpowered for GxE testing (Thomas, 2010) (median case sample size across the studies was 602; 428 within SCC studies, 602 melanoma, 732 BCC). Eleven of the included studies analysed only one or two genes in candidate gene approach, which limited the scope of genetic factors evaluated, and biases the field towards known skin cancer‐related genes. Additionally, some studies did not correct for multiple testing. This makes a comparison of what constitutes a significant finding across studies difficult. We did not conduct a meta‐analysis so significant findings were presented as reported in the original paper. Significance thresholds should be adjusted when testing multiple SNPs and should be interpreted carefully within the study context. While many studies used the same sample population, the evidence for GxE varied depending on the choice of genetic factor(s) and analysis set up, highlighting the effect study design decisions ultimately have on the findings.

## 5 CONCLUSION

Further GxE research in skin cancer is warranted, because it has potential to enable better understanding of skin cancer aetiology and the development of well‐informed personalized sun exposure recommendations. Adoption of a genome‐wide approach is recommended as it might uncover previously unknown disease pathways dependent on UVR.

## AUTHOR CONTRIBUTIONS

Lina Zgaga and Rasha Shraim were involved in conceptualization. Rasha Shraim was involved in methodology, writing—original draft preparation and visualization. Mohamed Ziad Farran, George He, Jelena Marunica Karsaj, Ross McManus and Rasha Shraim were involved in data curation. Rasha Shraim, Lina Zgaga and Ross McManus were involved in writing—review and editing. Lina Zgaga and Ross McManus were involved in supervision and should be considered joint senior author. All authors have read and agreed to the published version of the manuscript.

## CONFLICT OF INTEREST STATEMENT

The authors have no conflict of interest to declare.

## Supporting information


File S1.

File S2.

Table S1.

Table S2.

Table S3.

## Data Availability

Data sharing is not applicable to this article as no new data were created or analyzed in this study.
