# Supplementary material for: Systematic review on gene–sun exposure interactions in skin cancer
Source: Mol Genet Genomic Med. 2023 Aug 3;11(10):e2259. doi: 10.1002/mgg3.2259 (PMC10568388; doi:10.1002/mgg3.2259)
Supplement: Supplementary file 1 — File S1. [file MGG3-11-e2259-s005.docx]

**Search Strategy for GxE skin cancer by database**

EMBASE

1. 'genotype environment interaction'/exp
2. (gene* NEAR/3 environment* NEAR/3 interact*):ti,ab
3. (‘genotype × environment’ OR 'gene × environment' OR ‘G×E’ OR ‘Gene-environment correlation*’):ti,ab
4. #1 OR #2 OR #3
5. 'skin cancer'/exp OR 'melanoma'/exp OR 'skin tumor'/exp
6. (Skin NEAR/3 (cancer OR neoplasm* OR tumour* OR tumor* OR metastasis OR carcinoma* OR 'squamous carcinoma')):ti,ab
7. ('basal cell' NEAR/3 (carcinoma* OR epithelioma OR neoplasm* OR tumor* OR tumour* OR 'squamous carcinoma')):ti,ab
8. ('basalioma' OR 'basaloid tumor*' OR 'basaloid tumour*' OR 'basaloma*' OR 'basaloma terebrans' OR 'basocellular carcinoma*' OR 'basocellular epithelioma*' OR 'basosquamous carcinoma*'):ti,ab
9. (Melanoma* OR 'malignant melanomatosis' OR 'melanocarcinoma' OR 'melanoma' OR 'melanomalignoma' OR 'naevi and melanomas' OR 'naevocarcinoma' OR 'nevi and melanomas' OR 'nevocarcinoma' OR 'pigmentary cancer' OR ‘cutaneous metastasis’):ti,ab
10. (cutaneous NEAR/3 (cancer OR neoplasm* OR tumour* OR tumor* OR metastasis OR carcinoma* OR 'squamous carcinoma')):ti,ab
11. #5 OR #6 OR #7 OR #8 OR #9 OR #10
12. #4 AND #11

Medline (Ovid)

1. exp Gene-Environment Interaction/
2. (gene* ADJ3 environment* ADJ3 interact*).ti,ab.
3. (genotype environment or gene environment or Gene-environment correlation*).ti,ab.
4. 1 OR 2 OR 3
5. exp Skin Neoplasms/ OR exp Melanoma/ or exp Carcinoma, Basal Cell/
6. (skin cancer OR melanoma OR skin tumor).ti,ab.
7. (Skin ADJ3 (cancer OR neoplasm* OR tumour* OR tumor* OR metastasis OR carcinoma* OR squamous)).ti,ab.
8. (basal cell ADJ3 (carcinoma* OR epithelioma OR neoplasm* OR tumor* OR tumour* OR squamous)).ti,ab.
9. (basalioma OR basaloid tumor* OR basaloid tumour* OR basaloma* OR basaloma terebrans OR basocellular carcinoma* OR basocellular epithelioma* OR basosquamous carcinoma*).ti,ab.
10. (Melanoma* OR malignant melanomatosis OR melanocarcinoma OR melanoma OR melanomalignoma OR naevi and melanomas OR naevocarcinoma OR nevi and melanomas OR nevocarcinoma OR pigmentary cancer OR cutaneous metastasis).ti,ab.
11. (cutaneous ADJ3 (cancer OR neoplasm* OR tumour* OR tumor* OR metastasis OR carcinoma* OR squamous)).ti,ab.
12. or/5-11
13. 4 AND 12

Web of Science (Core Collection)

1. TS =("genotype environment interaction" OR (gene* NEAR/2 (environment* NEAR/2 interact*)) OR "genotype × environment" OR "gene × environment" OR "G×E" OR "Gene-environment correlation*") OR AB =("genotype environment interaction" OR (gene* NEAR/2 (environment* NEAR/2 interact*)) OR "genotype × environment" OR "gene × environment" OR "G×E" OR "Gene-environment correlation*")
2. TS=('skin cancer' OR 'melanoma' OR 'skin tumor') OR TS=(Skin NEAR/3 (cancer OR neoplasm* OR tumour* OR tumor* OR metastasis OR carcinoma* OR squamous)) OR TS=('basal cell' NEAR/3 (carcinoma* OR epithelioma OR neoplasm* OR tumor* OR tumour* OR squamous)) OR TS=('basalioma' OR 'basaloid tumor*' OR 'basaloid tumour*' OR 'basaloma*' OR 'basaloma terebrans' OR 'basocellular carcinoma*' OR 'basocellular epithelioma*' OR 'basosquamous carcinoma*') OR TS=(Melanoma* OR 'malignant melanomatosis' OR 'melanocarcinoma' OR 'melanoma' OR 'melanomalignoma' OR 'naevi and melanomas' OR 'naevocarcinoma' OR 'nevi and melanomas' OR 'nevocarcinoma' OR 'pigmentary cancer' OR ‘cutaneous metastasis’) OR TS=(cutaneous NEAR/3 (cancer OR neoplasm* OR tumour* OR tumor* OR metastasis OR carcinoma* OR squamous))
3. #1 AND #2
