# Supplementary material for: Systematic review on gene–sun exposure interactions in skin cancer
Source: Mol Genet Genomic Med. 2023 Aug 3;11(10):e2259. doi: 10.1002/mgg3.2259 (PMC10568388; doi:10.1002/mgg3.2259)
Supplement: Supplementary file 3 — Table S1. [file MGG3-11-e2259-s004.docx]

Table S1: Quality assessment based on the Newcastle-Ottawa Scale for cohort studies and for case-control studies. The score is shown out of 9 possible points for cohort and 8 possible point for case-control studies.

| **Cohort Scale** | Selection: Representativeness of the exposed cohort | Selection: Selection of the non exposed cohort | | Outcome: Assessment of outcome | | Comparability: Comparability of cohorts on the basis of the design or analysis | | Comparability: Comparability of cohorts on the basis of the design or analysis | | Selection: Ascertainment of exposure | | Selection: Demonstration that outcome of interest was not present at start of study | | Outcome: Was follow-up long enough for outcomes to occur? | | Outcome: Adequacy of follow up | **Score (9)** |
| --- | --- | --- | --- | --- | --- | --- | --- | --- | --- | --- | --- | --- | --- | --- | --- | --- | --- |
| **Olsen 2020** | somewhat representative (+) | same community as exposed (+) | | record linkage (+) | | controls for [sun exposure habits] (+) | | controls for any additional factor [age, sex, BMI] (+) | | secure record (+) | | N/A | | yes (+) | | unlikely to introduce bias (+) | **8** |
| **Case control Scale** | Selection: Is the case definition adequate? | | Selection: Representativeness of the cases | | Selection: Selection of Controls | | Selection: Definition of Controls | | Comparability | | Exposure: Ascertainment of exposure | | Exposure: Same method of ascertainment for cases and controls | | Exposure: Non-Response rate | | **Score (8)** |
| **Ng 2011** | ?? | | ?? | | ?? | | no description of source | | controls for [sun exposure habits] (+) | | no description | | ?? | | ?? | | **1** |
| **He 2010** | record linkage | | potential for selection biases or not stated | | community controls (+) | |  | | controls for [sun exposure habits] (+) | | no description | | yes (+) | | rate different and no designation | | **3** |
| **Lin 2017** | record linkage | | potential for selection biases or not stated | | community controls (+) | | no description of source | | controls for [sun exposure habits] (+) | | secure record (+) | | yes (+) | | rate different and no designation | | **4** |
| **Rizzato 2011** | independent validation (+) | | potential for selection biases or not stated | | hospital controls | | no history of disease (+) | | controls for any additional factor [age, sex, BMI] (+) | | interview not blinded to case/control status | | yes (+) | | ?? | | **4** |
| **Berwick 2010** | independent validation (+) | | representative (+) | | hospital controls | | no history of disease (+) | | controls for [sun exposure habits] (+) | | no description | | yes (+) | | rate different and no designation | | **5** |
| **Chahal 2016** | independent validation (+) | | potential for selection biases or not stated | | community controls (+) | | no history of disease (+) | | controls for [sun exposure habits] (+) | | secure record (+) | | yes (+) | | rate different and no designation | | **6** |
| **Kricker 2010** | independent validation (+) | | potential for selection biases or not stated | | community controls (+) | | no history of disease (+) | | controls for any additional factor [age, sex, BMI] (+) | | secure record (+) | | yes (+) | | rate different and no designation | | **6** |
| **Li 2007** | independent validation (+) | | representative (+) | | community controls (+) | | no history of disease (+) | | controls for [sun exposure habits] (+) | | interview not blinded to case/control status | | yes (+) | | rate different and no designation | | **6** |
| **Nelson 2002** | independent validation (+) | | representative (+) | | community controls (+) | | no history of disease (+) | | controls for [sun exposure habits] (+) | | interview not blinded to case/control status | | yes (+) | | rate different and no designation | | **6** |
| **Nelson 2005** | independent validation (+) | | representative (+) | | community controls (+) | | no history of disease (+) | | controls for any additional factor [age, sex, BMI] (+) | | interview not blinded to case/control status | | yes (+) | | rate different and no designation | | **6** |
| **Li 2006 (Pharmacogenetics & Genomics)** | record linkage | | representative (+) | | community controls (+) | | no history of disease (+) | | controls for [sun exposure habits] (+) | | interview not blinded to case/control status | | yes (+) | | same rate for both groups (+) | | **6** |
| **Li 2006 (Carcinogenesis)** | record linkage | | representative (+) | | community controls (+) | | no history of disease (+) | | controls for any additional factor [age, sex, BMI] (+) | | interview not blinded to case/control status | | yes (+) | | same rate for both groups (+) | | **6** |
| **Welsh 2008** | independent validation (+) | | representative (+) | | community controls (+) | | no history of disease (+) | | controls for [sun exposure habits] (+) | | interview not blinded to case/control status | | yes (+) | | same rate for both groups (+) | | **7** |
| **Mandelcorn Monson 2011** | independent validation (+) | | representative (+) | | community controls (+) | | no history of disease (+) | | controls for any additional factor [age, sex, BMI] (+) | | secure record (+) | | yes (+) | | same rate for both groups (+) | | **8** |
