# Supplementary material for: Systematic review on gene–sun exposure interactions in skin cancer
Source: Mol Genet Genomic Med. 2023 Aug 3;11(10):e2259. doi: 10.1002/mgg3.2259 (PMC10568388; doi:10.1002/mgg3.2259)
Supplement: Supplementary file 4 — Table S2. [file MGG3-11-e2259-s003.docx]

Table S2 :Additional information from the included studies: quality control measures for the genetic variants and other covariates considered in the analyses.

| **Citation** | **Gene (quality control)** | **Other covariates considered** |
| --- | --- | --- |
| **Ng 2011*** | MC1R variants |  |
| **Olsen 2020** | PGS (missingness, relatedness, population outliers, GenTrain score, HWE, MAF, imputation score) | Pigmentation, family history of melanoma, sun protection behaviors, education |
| **Li 2006** (Pharmacogenetics & Genomics) | FAS and FASLG combined genotypes (HWE) | Age, sex, and host characteristics (e.g. colour of skin, eyes and hair), sun exposure history, Fitzpatrick skin type, presence of moles/nevi, family history of cancer |
| **Li 2006** (Carcinogenesis) | APE1 and XRCC1 combined genotype (HWE) | Age, sex, and host characteristics (e.g. colour of skin, eyes and hair), sun exposure history, Fitzpatrick skin type, presence of moles/nevi, family history of cancer |
| **Li 2007** | nNOS and iNOS SNPs (HWE) | Age, sex, host characteristics, sun exposure hisotyr, Fitzpatrick skin type, moles/nevi, family history of cancer |
| **Kricker 2010** | MC1R gene | Age, sex, ancestry, age-sex interaction, study center, pigment score |
| **Berwick 2010**** | CDKN2A | Age, sex, study center, age-sex interaction, ability to tan |
| **Mandelcorn-Monson 2011** | VDR FokI and Bsml polymorphisms | Age, sex, age-sex interaction |
| **He 2010** | GPX1 and CAT genes (HWE) | Age, constitutional susceptibility score, family history of skin cancer, lifetime severe sunburns, sunlamp or tanning use, cumulative sun exposure in bathing suit, geographic region |
| **Rizzato 2011** | 14 SNPs in 10 cytokine genes (HWE in controls), 3 previously reported SNPs | Age, gender, recruitment center, three know BCC risk factors (skin complexion, sun exposure, skin response to sun) |
| **Chahal 2016** | 31 GWAS signnificant loci (MAF, imputation quality score, HWE, ethnicity) | Age, sex, population stratification |
| **Lin 2017** | 2,540 independent SNPs across VDR binding sites (linkage disequilibrium) | Age, sex, population stratification |
| **Nelson 2002** | XRCC1 (HWE) | Age, sex, tendency to sunburn, cumulative sun hours, hair color, eye color |
| **Nelson 2005** | XPC PAT allele (HWE) | Age, sex, tendency to sunburn |
| **Welsh 2008** | HAL locus as a binary variable with dominant inheritance (HWE) | Age, gender, skin pigmentation, lifetime severe sunburns |
