# Supplementary material for: Systematic review on gene–sun exposure interactions in skin cancer
Source: Mol Genet Genomic Med. 2023 Aug 3;11(10):e2259. doi: 10.1002/mgg3.2259 (PMC10568388; doi:10.1002/mgg3.2259)
Supplement: Supplementary file 5 — Table S3. [file MGG3-11-e2259-s001.docx]

Table S3: Summary of genes tested for gene-environment interactions in melanoma, BCC, or SCC.

| **Gene** | **Chromosome** | **Melanoma** | **BCC** | **SCC** |
| --- | --- | --- | --- | --- |
| FASLG | 1 | not sig | - | - |
| IL10 | 1 | - | not sig | - |
| IL1A | 2 | - | not sig | - |
| IL1B | 2 | - | not sig | - |
| IL1RN | 2 | - | not sig | - |
| GPX1 | 3 | not sig | - | - |
| XPC | 3 | - | not sig | not sig |
| CXCL8 | 4 | - | not sig | - |
| IL2 | 4 | - | not sig | - |
| IL12B | 5 | - | not sig | - |
| IL4 | 5 | - | not sig | - |
| TNF | 6 | - | not sig | - |
| IL6 | 7 | - | not sig | - |
| CDKN2A | 9 | not sig | - | - |
| FAS | 10 | not sig | - | - |
| CAT | 11 | * (CAT C-262T; history of severe sunburns) | not sig | not sig |
| HAL | 12 | - | * (severe sunburns) | * (severe sunburns) |
| IFNG | 12 | - | not sig | - |
| IL23A | 12 | - | * (rs79824801; cumulative sun exposure) | - |
| NOS1 | 12 | * (sunburns) | - | - |
| VDR | 12 | not sig | - | - |
| APEX1 | 14 | not sig | - | - |
| MC1R | 16 | * (early life UV on body & neck) | - | - |
| NOS2 | 17 | not sig | - | - |
| XRCC1 | 19 | not sig | not sig | * (difference in models with and without GxE) |
| PGS |  | * (country of birth & actinic lesions) |  |  |
